# Gram-Negative Bacteria Harboring Multiple Carbapenemase Genes, United States, 2012–2019

**DOI:** 10.3201/eid2709.210456

**Published:** 2021-09

**Authors:** D. Cal Ham, Garrett Mahon, Sandeep K. Bhaurla, Sam Horwich-Scholefield, Liore Klein, Nychie Dotson, J. Kamile Rasheed, Gillian McAllister, Richard A. Stanton, Maria Karlsson, David Lonsway, Jennifer Y. Huang, Allison C. Brown, Maroya Spalding Walters

**Affiliations:** Centers for Disease Control and Prevention, Atlanta, Georgia, USA (D.C. Ham, G. Mahon, J.K. Rasheed, G. McAllister, R.A. Stanton, M. Karlsson, D. Lonsway, J.Y. Huang, A.C. Brown, M.S. Walters);; Los Angeles County Department of Public Health, Los Angeles, California, USA (S.K. Bhaurla);; California Department of Public Health, Richmond, California, USA (S. Horwich-Scholefield);; Maryland Department of Health, Baltimore, Maryland, USA (L. Klein);; Florida Department of Health, Tallahassee, Florida, USA (N. Dotson)

**Keywords:** drug resistance, carbapenem-resistant Enterobacterales, carbapenemase, *Acinetobacter baumannii*, *Pseudomonas*, antimicrobial resistance, AMR, carbapenems, *Enterobacteriaceae*, United States, resistance genes

## Abstract

Reports of organisms harboring multiple carbapenemase genes have increased since 2010. During October 2012–April 2019, the Centers for Disease Control and Prevention documented 151 of these isolates from 100 patients in the United States. Possible risk factors included recent history of international travel, international inpatient healthcare, and solid organ or bone marrow transplantation.

Carbapenems have been standard treatments for multidrug-resistant gram-negative bacilli infections since 1985, when they were approved for clinical use in the United States (https://www.accessdata.fda.gov/drugsatfda_docs/label/2016/050587s074lbl.pdf). Carbapenem-resistant organisms (CROs) are a growing public health concern as carbapenemase-producing CROs become more common (*1*). Several recent reports describe CROs carrying multiple carbapenemase genes (multi-CPOs) (*2*–*8*). We describe multi-CPOs reported to the Centers for Disease Control and Prevention (CDC; Atlanta, GA, USA) during 2012–2019.

## The Study

CDC receives reports of carbapenemase-producing CROs from health departments, public health laboratories, healthcare facilities, and isolates sent to CDC for confirmatory testing. In 2016, CDC established the Antibiotic Resistance Laboratory Network (AR Lab Network), a national network of 55 public health laboratories that test carbapenem-resistant Enterobacterales (CRE), carbapenem-resistant *Pseudomonas aeruginosa* (CRPA), and carbapenem-resistant *Acinetobacter baumannii* (CRAB) isolates for carbapenemase genes.

We reviewed CDC and AR Lab Network reports of multi-CPOs identified during January 1, 2010–April 30, 2019. We defined a multi-CPO case as Enterobacterales, *Pseudomonas* spp., or *A. baumannii* isolated from any specimen source and carrying genes encoding >1 carbapenemase routinely tested for at CDC and the AR Lab Network (CRE, CRPA, and CRAB isolates were tested for *Klebsiella pneumoniae* carbapenemase [KPC], New Delhi metallo-β-lactamase [NDM], Verona integron-encoded metallo-β-lactamase [VIM], active-on-imipenem metallo-β-lactamase [IMP], and oxacillinase [OXA]-48–like β-lactamases; CRAB isolates also were tested for OXA-23, OXA-24/40, and OXA-58–like β-lactamases). Whole-genome sequencing (WGS) was conducted on a subset of isolates (Appendix). We defined an incident case as the first isolation of a unique organism–carbapenemase combination in each patient.

As part of routine public health investigations, health departments reviewed medical records and laboratory reports for patient demographic data and risk factors for exposure. We conducted descriptive analyses using SAS version 9.4 (https://www.sas.com) and calculated Pearson χ^2^ score using SPSS Statistics 21.0 (IBM, https://www.ibm.com).

During January 2010–April 2019, a total of 151 multi-CPO isolates, including those from 105 incident cases, were identified in 100 unique patients; the first case was identified in October 2012 (Table 1; Appendix Tables 1, 2). Among 89 (84.8%) incident cases reported since AR Lab Network testing began in 2017, a total of 15 were reported in 2017, 51 in 2018, and 23 in the first 4 months of 2019. Among the isolates tested through the AR Lab Network during 2017–2019, a total of 111/28,390 (0.391%) CRE, 5/19,609 (0.025%) CRPA, and 2/2,443 (0.082%) CRAB isolates harbored multiple carbapenemase genes; we included CRAB isolates tested only during January 2018–April 2019. Incident cases were reported in 29 US states and the District of Columbia. Enterobacterales accounted for 96 (91.4%) of the incident multi-CPO cases; in addition, 7 (6.7%) were *Pseudomonas* spp. and 2 (1.9%) were *A. baumannii*. Among 96 incident Enterobacterales cases, the most common (46; 47.9%) organism–gene combination was *K. pneumoniae* harboring *bla*_NDM_ and *bla*_OXA-48–like_. 

**Table 1:** Incident cases of gram-negative bacilli harboring multiple carbapenemase genes, United States, January 2012–April 2019*

Organism	Carbapenemase combinations								Total, N = 105
	NDM + OXA-48–like	KPC + NDM	KPC + VIM	NDM + VIM	KPC + OXA-48–like	NDM + IMP	NDM + OXA-23	NDM + OXA-48–like + VIM	
Enterobacterales	64	23	6	0	2	0	0	1	96
* Citrobacter freundii*	0	0	1	0	1	0	0	0	2
* Enterobacter cloacae*	0	8	1	0	0	0	0	0	9
* Escherichia coli*	17	3	0	0	0	0	0	0	20
* Klebsiella aerogenes*	0	0	1	0	0	0	0	0	1
* K. oxytoca*	0	0	1	0	0	0	0	0	1
* K. pneumoniae*	46	12	2	0	1	0	0	1	62
* Providencia rettgeri*	1	0	0	0	0	0	0	0	1
Pseudomonadales	0	0	1	4	0	2	2	0	9
* Pseudomonas aeruginosa*	0	0	1	3	0	2	0	0	6
* Pseudomonas fluorescens*	0	0	0	1	0	0	0	0	1
* Acinetobacter baumannii*	0	0	0	0	0	0	2	0	2

*IMP, active-on-imipenem metallo-β-lactamase; KPC, *Klebsiella pneumoniae* carbapenemase; NDM, New Delhi metallo-β-lactamase; OXA, oxacillinase; VIM, Verona integron-encoded metallo-β-lactamase.

 WGS was conducted on 46 isolates from incident cases, identifying 6 sequence types of *Enterobacter cloacae*, 9 of *Escherichia coli*, and 11 of *K. pneumoniae*. WGS identified 21 isolates harboring *bla*_NDM-1_, 16 harboring *bla*_NDM-5_, 16 harboring *bla*_OXA-181_, and 11 harboring *bla*_KPC-3_ (Appendix Table 2). In total, 8 incident cases were associated with 2 separate clusters at acute care hospitals.

The median age of patients at the time of multi-CPO identification was 63 years (range 2–94 years). Among 93 incident cases with available data, 62 (66.7%) occurred in patients who had traveled internationally in the 12 months before their incident culture. Among patients with a history of international travel, most (89.5%) had received inpatient healthcare while abroad. Association with international travel varied by carbapenemase combination; among 59 incident cases with available data that harbored *bla*_NDM_ and *bla*_OXA-48–like_, 47 (79.7%) occurred in patients who reported international travel; only 5/19 (26.3%; p<0.01) cases that harbored *bla*_KPC_ and *bla*_NDM_ occurred in patients who reported international travel. Among the 80 incident cases with available data, 14 (17.5%) occurred in patients with a history of solid organ or bone marrow transplantation before their incident culture (Table 2).

**Table 2:** Characteristics and exposures of incident cases of gram-negative bacilli harboring multiple carbapenemase genes, United States, January 2012–April 2019*

Characteristics and exposures	Enterobacterales†				*Pseudomonas *spp.,‡ KPC + VIM, NDM + VIM, or NDM + IMP	*Acinetobacter baumannii*, NDM + OXA-23	Total
	NDM + OXA-48§	KPC + NDM	KPC + VIM	KPC + OXA-48			
Total no. (%) cases	65 (100.0)	23 (100.0)	6 (100.0)	2 (100.0)	7 (100.0)	2 (100.0)	105 (100.0)
Region of specimen collection¶							
South	22/65 (33.8)	9/23 (39.1)	2/6 (33.3)	0	3/7 (42.9)	1/2 (50.0)	37/105 (35.2)
West	22/65 (33.8)	3/23 (13.0)	2/6 (33.3)	0	1/7 (14.3)	0	28/105 (26.7)
Northeast	14/65 (21.5)	5/23 (21.7)	0	0	2/7 (28.6)	0	21/105 (20.0)
Midwest	7/65 (10.8)	6/23 (26.1)	2/6 (33.3)	2/2 (100.0)	1/7 (14.3)	1/2 (50.0)	19/105 (18.1)
Location of specimen collection							
Acute care hospital	51/57 (89.5)	18/22 (81.8)	3/4 (75.0)	2/2 (100.0)	5/7 (71.4)	0	79/94 (84.0)
Outpatient facility	5/57 (8.8)	1/22 (4.5)	0	0	2/7 (28.6)	1/2 (50.0)	9/94 (9.6)
Long-term acute care hospital	0	1/22 (4.5)	1/4 (25.0)	0	0	1/2 (50.0)	3/94 (3.2)
Skilled nursing facility	0	2/22 (9.1)	0	0	0	0	2/94 (2.1)
Joint acute care hospital/ inpatient rehabilitation facility	1/57 (1.8)	0	0	0	0	0	1/94 (1.1)
Hospitalization in previous 12 mo, United States#	44/56 (78.6)	19/23 (82.6)	4/5 (80.0)	2/2 (100.0)	4/7 (57.1)	2/2 (100.0)	75/95 (78.9)
International travel in previous 12 mo**							
Yes	47/59 (79.7)††	5/19 (26.3)††	1/4 (25.0)	1/2 (50.0)	7/7 (100.0)	1/2 (50.0)	62/93 (66.7)
International inpatient healthcare‡‡	40/43 (93.0)	3/4 (75.0)	0/1	0/1	6/7 (85.7)	1/1 (100.0)	51/57 (89.5)
India	29/39 (74.4)	1/3 (33.3)		1/1 (100.0)	3/6 (50.0)	1/1 (100.0)	35/50 (70.0)
Other§§	5/39 (12.8)	2/3 (66.7)		0	2/6 (33.3)	0/1	9/50 (18.0)
Pakistan	3/39 (7.7)	0/3		0/1	0/6	1/1 (100.0)	4/50 (8.0)
Egypt	2/39 (5.1)	0/3		0/1	0/6	0/1	2/50 (4.0)
Vietnam	1/39 (2.6)	0/3		0/1	1/6 (16.7)	0/1	2/50 (4.0)
No	12/59 (20.3)	14/19 (73.7)	3/4 (75.0)	1/2 (50.0)	0/7	1/2 (50.0)	31/93 (33.3)
US hospitalization	11/12 (91.7)	12/14 (85.7)	3/3 (100.0)	1/1 (100.0)		1/1 (100.0)	28/31 (90.3)
Transplant recipient¶¶	11/48 (22.9)	4/17 (23.5)	0/5	1/2 (50.0)	1/6 (16.7)	0/2	17/80 (21.3)
Before incident case	8/11 (72.7)	4/4 (100.0)		1/1 (100)	1/1 (100.0)		14/17 (82.4)
Transplant to incident case, d, median (IQR)							44 (15–446)
After incident case	3/11 (27.3)	0/4		0/1	0/1		3/17 (17.6)
Incident case to transplant, d, median (IQR)							96 (28–188)
Type of transplant##							
Solid organ	11/11 (100.0)	2/4 (50.0)		0/1	0/1		13/17 (76.5)
Kidney	7/11 (63.6)	0/2					7/13 (53.8)
Liver	3/11 (27.3)	1/2 (50.0)					4/13 (30.8)
Lung	1/11 (9.1)	1/2 (50.0)					2/13 (15.4)
Bone marrow	0/11	2/4 (50.0)		1/1 (100.0)	1/1 (100.0)		4/17 (23.5)

*Values are no. cases/total no. in category (%) except as indicated. Three incident cases occurred in 3 patients who reported no international travel or hospitalization in the United States during the previous 12 mo (1 case of *E. coli* harboring *bla*_NDM_ and *bla*_KPC_, 1 case of *K. pneumoniae* harboring *bla*_NDM_ and *bla*_KPC_, and 1 case of *E. coli* harboring *bla*_NDM_ and *bla*_OXA-48-like_). Among these patients, 1 was a nursing home resident, 1 did not have additional information provided, and 1 had a spouse who had traveled to India and returned ≈1 mo before their incident case. Exposures are described for the 12 mo before identification of incident case. IMP, active-on-imipenem metallo-β-lactamase; KPC, *Klebsiella pneumoniae* carbapenemase; NDM, New Delhi metallo-β-lactamase; OXA, oxacillinase; VIM, Verona integron-encoded metallo-β-lactamase. †*Citrobacter freundii*, *Enterobacter cloacae*, *Escherichia coli*, *Klebsiella aerogenes*, *K. oxytoca*, *K. pneumoniae*, and *Providencia rettgeri* isolates. ‡*Pseudomonas aeruginosa* and *Pseudomonas fluorescens* isolates. §Includes 1 *K. pneumoniae* isolate harboring *bla*_NDM_, *bla*_OXA-48-like_, and *bla*_VIM_. ¶Based on census regions of residence (US Census Bureau, https://www2.census.gov/geo/pdfs/maps-data/maps/reference/us_regdiv.pdf). #Of 90 unique patients who contributed 95 incident cases with complete data. **Of 88 unique patients who contributed 93 incident cases with complete data. ††Significant difference; p<0.01. Exclusion of incident cases associated with an outbreak or cluster did not change this association: 47/56 (83.9%) incident cases harboring *bla*_NDM_ and *bla*_OXA-48-like_ occurred in patients who reported international travel, compared with 4/14 (28.6%; p<0.01) with *bla*_KPC_ and *bla*_NDM_. ‡‡Two patients reported international inpatient healthcare in 2 countries. §§One hospitalization in Bangladesh, 1 in Columbia, 1 in Iraq, 1 in Mexico, 1 in Nigeria, 1 in Tajikistan, 1 in Thailand, 1 in Turkey, and 1 in Yemen. ¶¶Solid organ or bone marrow transplants; of 75 unique patients who contributed 80 incident cases with complete data. ##Of includes 17 unique patients who contributed 17 incident cases with complete data.

Multi-CPOs in this convenience sample were identified in many states and included diverse organisms, sequence types, and carbapenemase gene combinations and variants, suggesting that clonal spread is not responsible for their emergence. Variants harboring *bla*_KPC-4_ and *bla*_NDM-4_, which are uncommon in the United States, were identified (*9*–*11*). Most incident cases of CROs harboring multiple carbapenemase genes occurred in patients who had a recent history of international travel and inpatient healthcare outside the United States; we also identified history of solid organ or bone marrow transplant as a potential risk factor.

Receiving healthcare abroad and, more recently, international travel without medical care are risk factors for acquiring carbapenemase-producing organisms among patients in the United States (*9*). However, in this study, one third of cases occurred in persons without known recent travel outside the United States. For some carbapenemase combinations, such as isolates harboring *bla*_KPC_ and *bla*_NDM,_ most cases occurred in patients who had not recently traveled internationally. In addition, identifying of facility clusters raises further concerns about dissemination of these multidrug-resistant organisms among healthcare facilities in the United States.

The emergence of multi-CPOs has clinical, laboratory testing, and public health implications. The ceftazidime/avibactam, meropenem/vaborbactam, and imipenem/cilastatin/relebactam combination therapies have increased treatment options for CREs that produce KPC and OXA-48–like carbapenemases; growth in the proportion of isolates that co-harbor *bla*_NDM_ jeopardizes the usefulness of these therapies. We noted 1 *P. aeruginosa* isolate harboring *bla*_NDM-1_ and *bla*_IMP-1_; this isolate was panresistant to all antimicrobial drugs tested (*12*). A high proportion (17.5%) of cases occurred among patients with history of solid organ or bone marrow transplantation before their index culture, a population for whom CRO infections are associated with worse outcomes than patients without transplants (*13*,*14*). In comparison, only 3.1% of patients with CRE reported to the Multi-Site Gram-Negative Surveillance Initiative at CDC during 2012–2019 had a history of transplant before their positive culture (*15*; I. See, CDC, pers. comm., 2021 Jan 19); whether multi-CPOs are emerging in this population requires careful monitoring. Finally, hierarchical testing algorithms, in which testing is halted after detection of an initial carbapenemase, might not identify additional, less common carbapenemases (e.g., hierarchical testing might not identify *bla*_VIM_ in an isolate with *bla*_KPC_ and *bla*_VIM_).

The first limitation of our analysis is that these data represent a passively reported convenience sample during a period in which multiple changes in testing practices, including the establishment of the AR Lab Network, occurred. For this reason, we cannot determine whether multi-CPOs became more common during the evaluation period. Second, CROs from patients with a history of healthcare abroad might have been selected for mechanism testing, biasing detection toward this risk factor; bias might have been more influential early in the investigation period, when testing resources were limited. Finally, this analysis did not systematically document outpatient healthcare exposures and residence in long-term care facilities, which also might be relevant sources of exposure; 1 case in this analysis was associated with invasive urologic procedures abroad (*7*).

## Conclusions

Multi-CPOs in healthcare facilities are an emerging concern in the United States. Although hospitalization outside the United States was the most common risk factor, we found a substantial proportion of cases that were probably acquired in healthcare facilities in the United States. Several measures might slow further spread. First, screening patients who were recently hospitalized outside the United States can help prevent additional introductions of carbapenemase genes not commonly found in the United States. Second, molecular testing to identify carbapenemase genes should not use hierarchical algorithms. Finally, when a multi-CPO is identified, public health officials should assess for potential transmission (https://www.cdc.gov/hai/containment/guidelines.html).

Appendix Additional information on gram-negative bacteria harboring multiple carbapenemase genes.
