# Supplementary material for: Gram-Negative Bacteria Harboring Multiple Carbapenemase Genes, United States, 2012–2019
Source: Emerg Infect Dis. 2021 Sep;27(9):2475–9. doi: 10.3201/eid2709.210456 (PMC8386808; doi:10.3201/eid2709.210456)
Supplement: Appendix — Additional information on gram-negative bacteria harboring multiple carbapenemase genes. [file 21-0456-Techapp-s1.pdf]

Article DOI: https://doi.org/10.3201/eid2709.2100456

Gram-Negative Bacteria Harboring Multiple Carbapenemase Genes, United States, 2012–2019

Appendix

Appendix Methods

Laboratory Methods for Molecular Detection of Carbapenemase Genes

Molecular detection of targeted carbapenemase genes was conducted using $\geq$1 PCR-based protocols and platforms, namely the Centers for Disease Control and Prevention (CDC)'s laboratory-developed and validated methods, Gene Xpert Carba-R (Cepheid, https://www.cepheid.com), ARM-D β-Lactamase (Streck, https://www.streck.com), and Verigene Gram-Negative Blood Culture System (Nanosphere, http://www.nanosphere.us). Laboratories used the modified carbapenem inactivation method (mCIM) or CarbaNP as phenotypic tests to determine whether an organism is carbapenemase-producing (https://arpsp.cdc.gov/resources/arln-psp-technical-appendix.pdf).

Changes in Laboratory Testing Protocols During Investigation Period

Beginning in January 2017, laboratories in the Antibiotic Resistance Laboratory Network tested for New Delhi metallo-β-lactamase, *Klebsiella pneumoniae* carbapenemase, Verona integron-encoded metallo- β-lactamase, active-on-imipenem metallo-β-lactamase, and oxacillinase-48–like β-lactamases. Not all laboratories initially tested all isolates for all gene targets. Some laboratories instituted hierarchical testing algorithms; if bla_{KPC} and bla_{NDM} were not detected, the isolates were tested for additional targets validated in their testing menu. If carbapenemase-producing (i.e., testing positive by the modified carbapenem inactivation method or CarbaNP) isolates were negative for all targets tested, the laboratories forwarded the isolate to their regional laboratory or CDC for additional characterization. In November 2017, CDC deployed PCR specific to additional bla_{IMP} variants not identified by Gene Xpert Carba-R. In November 2018, CDC deployed PCR that detects additional oxacillinases commonly associated

with *Acinetobacter* spp. (i.e., *bla*$_{OXA-23}$, *bla*$_{OXA-24/40}$, *bla*$_{OXA-58}$). After approval, these the assays were made available to the Antibiotic Resistance Laboratory Network and were validated and implemented at different times by laboratories in the network.

Whole-Genome Sequencing (WGS) Methods

Results were obtained from CDC and 4 state public health laboratories (SPHLs) that commonly performed WGS on carbapenemase-producing, carbapenem-resistant organisms harboring multiple carbapenemase genes. We report results on carbapenemase gene variants and sequence types determined by multilocus sequence typing (MLST).

WGS at SPHL 1

Isolates were extracted using the Qiagen DNeasy Blood & Tissue kit (QIAGEN, https://www.qiagen.com) and DNA libraries were prepared using the PulseNet Illumina Nextera XT protocol before being loaded on the Illumina MiSeq (Illumina, https://www.illumina.com). Carbapenemase variants were identified using ABRicate (https://github.com/tseemann/abricate). MLST was performed at CDC.

WGS at SPHL 2

DNA was extracted with the Qiagen DNeasy Blood & Tissue Kit (QIAGEN and libraries were prepared with the Illumina Nextera XT Kit and sequenced with a MiSeq version 3.0 600-cycle Kit (Illumina). Reads were assembled with CLC Genomics Workbench (QIAGEN), and assemblies were submitted to the ResFinder tool for antimicrobial resistance gene prediction or the MLST tool for in silico MLST.

WGS at SPHL 3

Genomic DNA was extracted from isolates using the DNeasy Blood & Tissue Kit on a QIAcube (QIAGEN). Sequence libraries were prepared using the Nextera XT DNA Sample Preparation Kit and sequenced on the Illumina MiSeq system (Illumina).

Raw Illumina reads were processed with Trimmomatic version 0.38 (*1*) and paired, 250 bp reads were then de novo assembled into contigs with SPAdes version 3.12.0 (*2*). Assembly quality was assessed using quantitative measurements, including BUSCO version 3.1.0 (*3*,*4*), before MLST with mlst v2.16.2 (https://github.com/tseemann/mlst) and AR gene identification with ABRicate version 0.8.13 (https://github.com/tseemann/abricate). Final analysis of the AR

genes in the genome assembly compared gene identification between the National Center for Biotechnology Information Bacterial Antimicrobial Resistance Reference Gene Database (*5*), ResFinder (*6*), and Comprehensive Antibiotic Resistance Database (*7*) to determine the best matches.

WGS at SPHL 4

DNA extraction was performed on the Magnapure 24 automated platform (Roche Molecular Systems, https://www.roche.com). Whole genome sequencing was performed using Illumina Miseq (Illumina). Following sequencing, de novo assembly was performed using CLC Genomics Workbench (QIAGEN), and the resistance gene profile is analyzed through CGE's Resfinder database. MLST was performed using the Linux-based program MLST by Torsten Seeman, which uses the available schemes found in pubmlst (https://github.com/tseemann/mlst).

WGS at CDC

Genomic DNA was extracted using Promega Maxwell 16 MDx Instrument and Maxwell 16 Cell Low Elution volume DNA Purification Kit (Promega Corporation, https://www.promega.com). WGS was performed using the Illumina MiSeq System and MiSeq Reagent version 2.0 kit, generating 2 × 250 paired-end reads (Illumina).

The WGS data was processed with the QuAISAR-H pipeline (Quality, Assembly, species Identification, Sequence typing, Annotation, Resistance mechanisms for Healthcare pathogens, https://github.com/DHQP/QuAISAR_singularity/). The pipeline includes species verification using pyani (*8*), identification of MLST using PubMLST definitions (*9*), and antibiotic resistance gene calling using GAMMA (https://github.com/rastanton/GAMMA) against a database constructed from the nonredundant entries in the ARG-ANNOT (*10*), NCBI AMRFinder (*5*), and ResFinder (*11*) antimicrobial resistance databases.

References

1. Bolger AM, Lohse M, Usadel B. Trimmomatic: a flexible trimmer for Illumina sequence data. Bioinformatics. 2014;30:2114–20. PubMed https://doi.org/10.1093/bioinformatics/btu170
2. Bankevich A, Nurk S, Antipov D, Gurevich AA, Dvorkin M, Kulikov AS, et al. SPAdes: a new genome assembly algorithm and its applications to single-cell sequencing. J Comput Biol. 2012;19:455–77. PubMed https://doi.org/10.1089/cmb.2012.0021

3. Simão FA, Waterhouse RM, Ioannidis P, Kriventseva EV, Zdobnov EM. BUSCO: assessing genome assembly and annotation completeness with single-copy orthologs. Bioinformatics. 2015;31:3210–2. PubMed https://doi.org/10.1093/bioinformatics/btv351

4. Waterhouse RM, Seppey M, Simão FA, Manni M, Ioannidis P, Klioutchnikov G, et al. BUSCO applications from quality assessments to gene prediction and phylogenomics. Mol Biol Evol. 2018;35:543–8. PubMed https://doi.org/10.1093/molbev/msx319

5. Feldgarden M, Brover V, Haft DH, Prasad AB, Slotta DJ, Tolstoy I, et al. Validating the AMRFinder tool and resistance gene database by using antimicrobial resistance genotype-phenotype correlations in a collection of isolates [Erratum in: Antimicrob Agents Chemother. 2020;64:e00361–20]. Antimicrob Agents Chemother. 2019;63:e00483–19. PubMed https://doi.org/10.1128/AAC.00483-19

6. Zankari E, Hasman H, Cosentino S, Vestergaard M, Rasmussen S, Lund O, et al. Identification of acquired antimicrobial resistance genes. J Antimicrob Chemother. 2012;67:2640–4. PubMed https://doi.org/10.1093/jac/dks261

7. Jia B, Raphenya AR, Alcock B, Waglechner N, Guo P, Tsang KK, et al. CARD 2017: expansion and model-centric curation of the comprehensive antibiotic resistance database. Nucleic Acids Res. 2017;45:D566–73. PubMed https://doi.org/10.1093/nar/gkw1004

8. Pritchard L, Glover RH, Humphris S, Elphinstone JG, Toth IK. Genomics and taxonomy in diagnostics for food security: soft-rotting enterobacterial plant pathogens. Anal Methods. 2016;8:12–24. https://doi.org/10.1039/C5AY02550H

9. Jolley KA, Bray JE, Maiden MCJ. Open-access bacterial population genomics: BIGSdb software, the PubMLST.org website and their applications. Wellcome Open Res. 2018;3:124. PubMed https://doi.org/10.12688/wellcomeopenres.14826.1

10. Gupta SK, Padmanabhan BR, Diene SM, Lopez-Rojas R, Kempf M, Landraud L, et al. ARG-ANNOT, a new bioinformatic tool to discover antibiotic resistance genes in bacterial genomes. Antimicrob Agents Chemother. 2014;58:212–20. PubMed https://doi.org/10.1128/AAC.01310-13

11. Bortolaia V, Kaas RS, Ruppe E, Roberts MC, Schwarz S, Cattoir V, et al. ResFinder 4.0 for predictions of phenotypes from genotypes. J Antimicrob Chemother. 2020;75:3491–500. PubMed https://doi.org/10.1093/jac/dkaa345

Appendix Table 1. Specimen sources of incident cases of gram-negative bacilli harboring multiple carbapenemase genes, United States, January 2012–April 2019*

	Carbapenemase combinations								
Source	NDM + OXA-48–like	KPC + NDM	KPC + VIM	NDM + VIM	KPC + OXA-48–like	NDM + IMP	NDM + OXA-23	NDM + OXA-48–like + VIM	Total, N = 105
Urine	37	8	3	3	0	2	1	1	55
Blood	7	3	1	1	1	0	0	0	13
Respiratory	5	6	2	0	0	0	0	0	13
Rectal swab	5	5	0	0	0	0	1	0	11
Other†	6	0	0	0	0	0	0	0	6
Wound	2	1	1	0	0	0	0	0	4
Peritoneal fluid	1	0	0	0	1	0	0	0	2
Unknown	1	0	0	0	0	0	0	0	1

*IMP, active-on-imipenem metallo-β-lactamase; KPC, *Klebsiella pneumoniae* carbapenemase; NDM, New Delhi metallo-β-lactamase; OXA, oxacillinase; VIM, Verona integron-encoded metallo-β-lactamase.
†Comprises 1 sample from abdomen, 1 from an abscess, 1 from penile exudate, 1 from a foot, 1 from the peritoneal cavity, and 1 from pleural fluid.

Appendix Table 2. Sequence types and gene variants of incident cases of gram-negative bacilli harboring multiple carbapenemases, United States, January 2012–April 2019*

	Carbapenemase combinations (no.)					
STs and gene variants†	NDM + OXA-48–like	KPC + NDM	KPC + VIM	NDM + VIM	NDM + IMP	NDM + OXA-48–like + VIM
Enterobacterales						
Enterobacter cloacae						
ST78		KPC-4, NDM-1 (1)				
ST91		KPC-3, NDM-7 (1)				
ST114		KPC-3, NDM-1 (1)				
ST171		KPC-3, NDM-1 (1)				
ST597		KPC-4, NDM-7 (2)				
ST729		KPC-3, NDM-1 (2)				
Escherichia coli						
ST2	NDM-5, OXA-181 (1)					
ST39		KPC-3, NDM-4 (1)				
ST167/2		KPC-4, NDM-5 (1)				
ST361	OXA-181 (1)‡					
ST398		KPC-3, NDM-5 (1)				
ST635	NDM-5, OXA-181 (1)					
ST648	NDM-7, OXA-181 (1)					
ST940	NDM-7, OXA-181 (1)					
ST8346	NDM-5, OXA-181 (1)					
Klebsiella pneumoniae						
ST14	NDM-1, OXA-232 (1)					
ST15	NDM-1, OXA-181 (1); NDM-1 (1)§	KPC-3, NDM-1 (1)				
ST16	NDM-5, OXA-181 (1)					

STs and gene variants†	NDM + OXA-48–like	KPC + NDM	KPC + VIM	NDM + VIM	NDM + IMP	NDM + OXA-48–like + VIM
	Carbapenemase combinations (no.)					
ST147	NDM-5, OXA-181 (4); NDM-5, OXA-232 (2)	KPC-3, NDM-1 (1)				
ST163		KPC-3, NDM-1 (1)				
ST231	NDM-1, OXA 181 (2); NDM-5, OXA-232 (1)					
ST395	NDM-5, OXA-232 (1)					
ST859	NDM-1, OXA-181 (1)					
ST983	NDM-5, OXA-48 (1)					
ST2497	NDM-1, OXA-232 (1)					
ST3392	NDM-1, OXA-232 (1)					
Novel			KPC-2, VIM-4 (1)			NDM-5, OXA-232, VIM-2 (1)
Unknown		KPC-3, NDM-1 (1)	KPC-2, VIM-4 (1)			
Providencia rettgeri						
Unknown	NDM-1, OXA-181 (1)					
Pseudomonadales						
Pseudomonas aeruginosa						
ST244					IMP-1, NDM-1 (1)	
Unknown					IMP-15, NDM-1 (1)	
Pseudomonas fluorescens (unknown ST)				NDM-1, VIM-2 (1)		

*Three *K. pneumoniae* incident cases harboring *bla*$_{\text{NDM}}$ and *bla*$_{\text{OXA-48-like}}$ were associated with an outbreak at an acute care hospital. Of the 3 isolates, 2 underwent WGS; they were identified as ST147 harboring *bla*$_{\text{NDM-5}}$ and *bla*$_{\text{OXA-181.}}$ In a separate cluster at an acute care hospital, an additional 5 incident cases harboring *bla*$_{\text{KPC}}$ and *bla*$_{\text{NDM}}$ (3 *E. cloacae* and 2 *K. pneumoniae*) were identified during a 16-mo period. WGS of 4 of these 5 isolates demonstrated that all harbored *bla*$_{\text{KPC-3}}$ and *bla*$_{\text{NDM-1}}$ and that 2 isolates were *E. cloacae* ST729, 1 was *E. cloacae* ST114, and 1 was *K. pneumoniae* ST163. Excluding these 8 cases linked to clusters, 97 incident cases were identified in 29 US states and the District of Columbia; only 19 (19.6%) incident cases with the same organism–mechanism combination were identified from the same state within the same 90-d period, making it unlikely that small outbreaks were the primary cause of cases. IMP, active-on-imipenem metallo-β-lactamase; KPC, *Klebsiella pneumoniae* carbapenemase; NDM, New Delhi metallo-β-lactamase; OXA, oxacillinase; ST, sequence type; VIM, Verona integron-encoded metallo-β-lactamase; WGS, whole-genome sequencing.

†Determined by WGS.

‡WGS did not detect *bla*$_{\text{NDM}}$. The mobile genetic element carrying *bla*$_{\text{NDM}}$ might have been lost before sequencing, which was conducted ≈3–5 wks after initial characterization.

§WGS did not detect *bla*$_{\text{OXA-48-like}}$. The mobile genetic element carrying *bla*$_{\text{OXA-48-like}}$ might have been lost before sequencing, which was conducted ≈3–5 wks after initial characterization.
